# Towards ethical drug pricing: the European Orphan Genomic Therapies Fund

**DOI:** 10.1038/s41434-024-00452-2

**Published:** 2024-04-24

**Authors:** Johanna Risse, Merlin Krzemien, Jan Schnalke, Thomas Heinemann

**Affiliations:** Institute for Medical Humanities, University Hospital Bonn, Venusberg-Campus 1, D-53127 Bonn, Germany

**Keywords:** Diseases, Genetics

## Abstract

An increasing number of novel genomic therapies are expected to become available for patients with rare or ultra-rare diseases. However, the primary obstacle to equal patient access to these orphan genomic therapies are currently very high prices charged by manufacturers in the context of limited healthcare budgets. Taking into account ethical pricing theories, the paper proposes the implementation of a pricing infrastructure covering all European member states, which has the potential to promote distributive justice while maintaining the attractiveness of genomic therapy development.

Novel methods like CRISPR-Cas and RNA technologies vastly expand the possibilities for targeted interventions in genomic functions, thus extending the concept of classical gene therapy towards novel ‘genomic therapies’. These therapies offer hope to patients who currently lack effective treatment options for severe or life-threatening genome-based disorders by precisely targeting disease-causing gene expression at various genomic levels. The recent scientific advances are particularly game-changing for patients with rare or ultra-rare diseases (less than 5 out of 10,000 people [1] and less than 1 out of 50,000 people [2], respectively). About 80 per cent of these disorders were annotated with one or more inheritance patterns which make them particularly susceptive to genomic intervention [3]. The promise of these interventions is a lasting therapeutic benefit with a ‘one-and-done’ treatment instead of a lifetime of chronic disease management [4]. However, the very high prices charged by pharmaceutical companies for these breakthrough therapeutics may ultimately put them out of reach of those patients who urgently need them [5–7]. For example, Hemgenix, a factor IX haemophilia genomic therapy developed by CSL Behring, costs around $3.5 million and is currently the world’s most expensive drug [8]. Other record-breaking prices have even led to some therapies being withdrawn from the market after price negotiations were unsuccessful [9–11]. This situation raises two ethical questions: First, is it morally wrong to charge such high prices for potentially life-saving drugs? Second, what would be an ethical and economically sound pricing process which increases equal access to novel genomic therapies for rare and ultra-rare diseases?

Ethics of pricing classically invoke market mechanisms. For example, the philosopher Alan Wertheimer discusses the idea of a fair and non-exploitative pricing process in his influential book ‘Exploitation’. He asserts that a fair and non-exploitative price would be determined in a competitive market and provides both an economic explanation and ethical justifications. The economic explanation is that in a competitive market, both sellers and buyers are price-takers, who have no control over the price and must accept the market-determined price. The ethical justification relates to the moral dimension of the relationship between buyer and seller: in a competitive market, “neither party takes special unfair advantage of particular defects in the other party’s decision-making capacity or special vulnerabilities in the other party’s situation” [12]. However, while this situation is characteristic for an ideal market situation, in the field of novel genomic therapies the situation is significantly different: Firstly, as essential or life-saving therapies, they are ‘priceless goods’ [13], defined as goods that are widely perceived to have a special non-market value. Secondly, the patients receiving these therapies are highly reliant on the treatment, and thus depend on healthcare providers to pay for them. Thirdly, the sellers are companies that, in the absence of a competitive market, enjoy a monopoly on their products, allowing them to demand whatever reimbursement and reward they believe is appropriate for their innovative therapy. In conclusion, the pricing of innovative genomic therapies seems to be exploitative and unfair based on Wertheimer’s ethics of pricing: Without a competitive market, pharmaceutical companies can take advantage of patients’ struggles and engage in price gouging, raising prices far above production and investment costs.

So where do we go from here?

The philosopher Matt Zwolinski presents a contrasting perspective on the issue of price gouging. He examines the issue of raising prices during emergency situations. However, his rationale against the commonly held intuition that price gouging is always an instance of wrongful exploitation also applies to pharmaceutical pricing. He calls it into question by revealing a philosophical conundrum: If price gouging ultimately improves the situation of desperate people in need by providing the needed good, how can such action be morally wrong, particularly if many of us do nothing to help people in need and generally feel no sense of wrongdoing for failing to do so? Zwolinski’s response: “On the face of it, those who ignore the needs of the vulnerable altogether treat them with *less* respect than those who do *something* to help” [14]. Additionally, he states that price gouging can be moral if it revitalises the economy and drives innovation.

Zwolinski’s arguments lead us to a crucial aspect of the ethics pertaining to the pricing of novel genomic therapies: the beneficial effects and moral advantages of price gouging *for the patient*. Today, the majority of these therapies target rare and ultra-rare diseases and therefore are designated as ‘orphan medicinal products’ [15]. In the past, manufacturers have given such products and thus rare diseases less attention due to the small patient populations making them commercially unattractive [16]. Moreover, for patients suffering from ultra-rare diseases with a mutation unique to them (*an N of 1*) or a mutation known to cause the disease in fewer than 30 patients worldwide, personalised genomic therapies based on their individual genetic profile are needed [17]. Under the current reimbursement system, personalised treatments for rare and ultra-rare diseases provide an economic challenge for drug manufacturers. Because of high up-front research and development (R&D) costs, manufacturers intend to recover these expenses by achieving a profit. However, with limited potential financial gains in a competitive market, producing orphan medicinal products may not justify the costs of R&D. In particular, the development of a novel genomic therapy is a very time-consuming and expensive process, according to the Innovative Genomics Institute [18]. To encourage development, many countries have introduced orphan drug exclusivity (ODE) and other regulatory, commercial, and financial incentives by law. Patenting in general is widely considered to be a major incentive for high-risk, high-cost medical innovation, because it provides a government-granted legal monopoly for the duration of the patent, theoretically allowing the patent holder to charge any price, regardless of its actual cost. ODE goes beyond this kind of protection by offering another lucrative advantage to manufacturers: It provides protection against the approval of another application for the same orphan indication. For example, the US Orphan Drug Act (1983) grants ODE for seven years [19], and the European Regulation on Orphan Medicinal Products (1999) grants ODE for 10 years [1]. In this regard, the government-granted monopolies have been successful: 244 designated orphan medicinal products with EU-wide marketing authorisations have been approved since the orphan legislation was introduced [20]. Due to these developments, pharmaceutical companies now consider orphan drugs to be ‘niche busters’ – treatments for small target groups whose high price make them as financially attractive as the ‘blockbuster drugs’ sold at lower price point to the masses [21]. With an estimated 95 per cent of over 7 000 rare disorders lacking approved treatment, numerous innovative genomic therapies are anticipated [22]. In summary, recent technological advancements in genomic research, combined with regulatory incentives have led to the development of a unique class of medicines that pose new economic and ethical challenges [23]: *orphan genomic therapies*. These therapies are unique in that they target very small patient populations with high unmet medical needs and aim to cure the disease with a single or only few numbers of treatments rather than providing lifelong chronic management – but at unaffordable prices.

However, the commercialisation of orphan genomic therapies varies greatly in terms of success. For example, Zolgensma, a genomic therapy for spinal muscular atrophy, is marketed globally by Novartis (Basel, Switzerland), despite its list price of $2.1 million. In 2022, the therapy generated a profit of $1.4 billion [24]. By comparison, BlueBird Bio (Somerville, Massachusetts) has withdrawn its operations for the treatment of beta-thalassemia with the genomic therapy Zynteglo from the European market due to “difficulties in achieving satisfactory value recognition and market access” [25]. This decision was made after negotiations in Germany failed regarding the company’s price tag of $1.8 million, causing BlueBird Bio to shift their focus to the US market. Therefore, Zynteglo is currently available only in Qualified Treatment Centers in the USA. Another example of failed commercialisation of an orphan genomic therapy is provided by Strimvelis, so far the only curative treatment with market authorisation in Europe for the rare primary immunodeficiency syndrome ADA-SCID. Orchard Therapeutics (London, UK), the biopharmaceutical company that marketed Strimvelis, has announced that it can no longer sustain sales due to a lack of profitability. The reasons for the commercial failure of Strimvelis are complex and might also hinge on reservations against the drug due to malignancies associated with its retroviral vector [26], although an alternative based on a lentiviral vector [27] has so far not succeeded in terms of commercialisation either. The licence to produce and distribute Strimvelis has since been purchased by the Telethon Foundation, but accessing the medication remains still difficult for patients as it is presently offered solely in Milan, Italy [28]. These examples illustrate that broad access to orphan genomic therapies not only depends on reimbursement decisions, but also on other factors like successful commercialisation and the geographical location of the patient. In addition, the pricing of orphan genomic therapies is of particular concern for low- and middle-income countries (LMICs). While genomic therapy research, development and clinical testing is mainly limited to high-income countries, some rare disorders are more common in other regions of the world [29]. For instance, while sickle cell anaemia (SCA) is a rare disease in Europe, a substantial number of SCA-patients live in sub-Saharan Africa. However, Casgevy, the first CRISPR-Cas9-based medicinal product for SCA, which was recently approved in the UK, EU and US [30], is likely to be out of reach for African patients due to its expected high price and the specialist centres required for treatment.

However, patents and market exclusivity rights are often viewed as the primary incentive for innovation and filling therapeutic gaps, not only for orphan genomic therapies but also other high-priced innovative medicines. Nevertheless, this system is also seen as being exploited by pharmaceutical companies for excessive profits [31]. As a result, policymakers, academic economists, patient organisations, pharmaceutical industry executives and ethicists are debating the fairness of these high prices [32]. For example, Hans Steutel, representing the German Association of Research-Based Pharmaceutical Companies, argues that it would be “scientifically impossible to assess drug prices according to criteria of fairness and justice” [33]. It would also be impossible to measure drug prices according to development, production, and distribution costs. However, Steutel’s view stands in contrast to a broad body of literature that proposes algorithms or methods for determining a fair or reasonable price for innovative medicine [34–36]. These fair pricing models are suggested as an alternative to the current patent and market exclusivity system. They are known as ‘cost-based pricing’ [37], as they aim to re-establish the connection between the price and the actual costs of developing and producing medicines, while allowing for a ‘fair profit margin’ for the companies. Although different parameters are used, the common objective is to achieve price transparency, which is believed to contribute to expanded access to innovative medicines by reducing their prices. However, several practical problems and disadvantages of these models have been discussed in the context of rare or ultra-rare diseases. For example, there is currently no widely agreed method for calculating costs or defining a fair profit for orphan genomic therapies [38, 39]. Additionally, the impact of transparency measures on accessibility of orphan genomic therapies remains largely theoretical and controversial. A major concern is that enforced transparency on costs and price-setting may “have a reverse effect on access and affordability” [40]. This is because manufacturers may abandon differential pricing schemes and apply uniform pricing for all countries, including LMICs [41, 42]. Moreover, a key concern with a cost-based pricing approach are “inherent risks of unwanted stakeholder behaviour, by both industry and payers” [43], such as a misallocation of rewards that could incentivise inefficient R&D without providing sufficient incentives to innovate medicines with social value. In other words, cost-based pricing may not adequately incentivise the development of drugs that offer health gains to patients and savings to the healthcare system and society [44].

Steutel as well as the European Federation of Pharmaceutical Industries and Associations are advocating for another pricing approach of innovative drugs [45], which competes with cost-based pricing: so-called ‘value-based pricing’. This approach suggests that the price of a new drug should be based on the additional value it brings to patients and society compared to conventional therapies (‘net incremental benefits’) [37]. However, at least at the time of approval, this approach does not work with orphan genomic therapies: Due to the limited number of rare disease patients scattered around the world, traditional clinical trials are not feasible. This leads to significant uncertainty at the time of marketing authorisation concerning how many patients will benefit, to what extent and over which time span [21]. For the same reason, clinical effect size remains unpredictable, leading to payers reimbursing treatments without sufficient knowledge of their benefits and harms. The high level of uncertainty, the high prices of orphan genomic therapies, and the significant unmet medical needs of patients present a challenging situation for payers, which applies to cost-based pricing and value-based pricing models alike.

This situation brings us back to the first question we raised at the beginning of this paper. Is it morally wrong to charge such high prices for potentially life-saving drugs? Based on the arguments outlined above, the answer is that the charging of high prices is not *inherently morally wrong*. We can now say that this is because in the issue of pricing there are two conflicting moral goods at stake. At the level of pharmaceutical R&D, ODE regulations and expensive rewards for orphan genomic therapies *work against inequalities* for people with complex, personalised health needs who are neglected by the competitive market incentive system. On the level of accessing these therapies, these incentives *promote inequalities* as they cannot be sustained by the highly limited resources of the healthcare system. The complexity of the ethical argument arises from the fact that these goods seem to cancel each other out: Genomic therapy research is worthless to the patient in need if the product is unaffordable, and lower prices are also worthless to the patient in need in the long term if they make the development of such therapies unattractive to companies. This presents a dilemma as it is difficult to weigh between goods which are not independent of each other.

Against this background, the answer to the question of whether it is morally wrong to charge such high prices for potentially life-saving drugs can be reformulated more straightforwardly: It is not the act of charging a high price for orphan genomic therapies per se that has moral implications, but rather its positive and negative effects on distributive justice. In his ground-breaking work ‘A Theory of Justice’, the philosopher John Rawls contends that constructing a society where the individuals’ lives are simply left to the outcome of the ‘natural lottery’ is unjust [46]. These morally arbitrary natural inequalities include the disadvantages caused by the high mortality and morbidity rates of genetic diseases [47]. An alternative way of structuring society is possible through distributive justice, that is, a system of formal equality of opportunity. In the field of healthcare, competing theories of distributive justice strive to demonstrate the fundamentals of equality of opportunity and how this principle can be achieved in situations of scarcity. In this regard, the philosopher Norman Daniels provides a highly influential account, highlighting that health represents a unique social good as it is central to the achievement of other goods. Daniels believes that health is a precondition for equal opportunities. Consequently, he considers distributive justice in healthcare as the ‘fair equality of opportunity’. It follows that “there should be no obstacles – financial, racial, geographical, and so on – to access the basic tier of the system” [48]. Orphan genomic therapies, if they are successful, are pertinent to this basic tier as they have the potential to cure, extend lifetime or increase quality of life for patients who were earlier deemed untreatable. However, given the limited budget, the growing number of orphan diseases and the predicted exponential increase of potential orphan genomic therapies, healthcare systems will not be able to cover the costs without undermining the rights of patients who suffer from common diseases. In any case, inequalities in access to health care are inevitable, and even more so for uninsured patients who will never be able to afford these therapies. But the potential positive effects on distributive justice of innovations such as orphan genomic therapies are often overlooked [49]: their opportunities to reduce unjust disadvantages arising from the natural lottery.

This leads us to the second question posed at the beginning of this paper: What would an ethical pricing process that would increase equitable access to orphan genomic therapies look like, as an alternative to the current patent and market exclusivity system? In the realisation of distributive justice, the applied procedures are seen to play an essential role, according to Rawls. He offers several examples in his ‘Theory of Justice’ to demonstrate how the process itself can influence the desired outcome. One well-known example is that of a number of people being asked to divide a cake: Rawls suggests that the optimal method would be to ensure the person cutting the cake takes the final piece, thus ensuring equal portions for all parties involved [46]. This example also illustrates how profound the impact of the procedure can be on the desired outcome. Can procedures also play a central role in achieving the desired outcome of equal access to orphan genomic therapies, while incentivising genomic research?

A widely discussed approach as an alternative to the standard reimbursement processes, which are currently based on up-front, single payments, are outcome-based agreements [50]. For example, a performance-based reimbursement structure can involve paying for genomic therapies at rates over a number of years that are adjusted according to the real-world outcomes of the treatment [51]. They can reduce the financial risk to payers, as manufacturers will share the costs if the treatment fails or is considered underperforming. These agreements vary in the evidence of benefit assessed (at population, individual, or trial level), payment terms (coverage with evidence or payment-by-results), and logistical details [52]. Luxturna, for instance, is an orphan genomic therapy approved for the curative treatment of patients with loss of vision due to inherited retinal dystrophy. To share the risk if patient outcomes fail to meet a specified threshold, Spark Therapeutics, the manufacturer, introduced an outcome-based rebate scheme. This scheme requires Luxturna to demonstrate its short-term efficacy (within 30–90 days) and long-term durability (30 months) through an eyesight assessment for each treated patient [50].

Although outcome-based agreements have been used for other orphan genomic therapies, such as Kymriah, Yescarta and Zolgensma, the degree to which payers, developers and healthcare providers are open to embrace innovative contracting varies across Europe [52]. This is due to various practical difficulties such as the agreement of financial terms in the context of 12-month budget cycles, potential violations of relevant international accounting rules, the absence of clear governance structures, and the need for additional data collection including the resulting administrative burden and costs [53]. For example, in Germany, the Federal Government has criticised pay-for-performance contracts, arguing that the task of collecting and evaluating patient outcomes would place a huge administrative burden on health insurers, both in terms of drafting and implementing the contracts and evaluating the data, and could therefore lead to high transaction costs [54]. Another unresolved issue concerns the case when patients switch their health insurance, rendering pay-for-performance contracts ineffective. For pharmaceutical companies, these models also present a complex challenge, as they must negotiate individual reimbursement agreements and outcome milestones with each country or insurer separately. This can take considerable time and effort to negotiate, especially in the European context with 27 countries [52].

Against the backdrop of these obstacles, it is crucial that novel contracting models such as pay-for-performance schemes are linked to an infrastructure spanning all European member states. In the case of rare diseases, the benefits of centralising the involved administrative tasks are particularly apparent: considering the low numbers of therapy applications, pooling evaluation is clearly more efficient than many times replicating a structure at national level. In a related context, the philosopher Thomas Pogge and economist Aidan Hollis propose the introduction of a mechanism that creates incentives for health outcomes instead of patent-protected mark-ups, with the aim of striking a balance between research incentives and global patient access to life-saving medicines, and therefore enhancing justice [55]. This mechanism is named the Health Impact Fund (HIF), a government-funded pay-for-performance mechanism. Pharmaceutical companies that register with the HIF agree to make their products available at the lowest feasible cost of production and distribution for a defined period. During this time, they receive annual payments based on the impact of their product on global health, as estimated through a global health impact assessment. The HIF, as described by Pogge and Hollis, pledges to distribute initially $6 billion per year to registered products in proportion to their share of the annually assessed health impact of all products registered with the HIF. This approach would allow the annual reward pools to be increased with the aim of attracting a larger share of innovative medicines.

The outcome-based system implemented by the HIF is an ethically defensible pricing mechanism as it increases distributive justice and addresses the dilemma between patient access and research incentives. The central concept is that healthcare providers and payers can purchase medicines from the fund at a very low price, allowing to provide *immediate access* to patients in need around the world: “The Health Impact Fund would give companies incentives to develop new products targeting the diseases and conditions for which existing systems have failed to produce results, which would especially benefit the poor” [56]. Since the HIF would not specifically address orphan genomic therapies, we see the need to establish an European Orphan Genomic Therapies Fund (EOGTF), which could help to balance the need to support further research and development with the commitment to *immediate and universal* access at the European level. Because this approach separates the incentive for innovations from the pricing of access, it reconciles to some extent the competing approaches of cost-based and value-based pricing, while avoiding their risks and drawbacks: Access is based on R&D costs, while reward reflects the real-world value their product delivers, leading to transparency in the pricing process. The main strength of this model is that, firstly, it incentivises companies to bring only orphan genomic therapies with therapeutic efficacy to market. Secondly, payers can make noteworthy savings as the EOGTF only pays the incentive once the long-term benefits have been established in the individual patient, thus bypassing the costs and burden of traditional therapies. Moreover, the EOGTF would be particularly beneficial in terms of savings if the therapy provided by the manufacturer did not show the expected outcome, in contrast to cost-based pricing models. Thirdly, the significant administrative burden of measuring long-term outcomes falls entirely on one organisation, and companies no longer need to engage in lengthy negotiations with individual countries. In addition, the EOGFT is likely to have more influence than individual companies or countries on the implementation of a global perspective of distributive justice with regard to access to orphan genomic therapies. For LMICs, the issue of access is different, as they lack not only the financial and manufacturing capacity of high-income countries to produce orphan genomic therapies, but also the bargaining power to buy these drugs at affordable prices. If the commercialisation of orphan genomic therapies is successful at the European level, then it is to be expected that it will also improve access to these innovative medicines on a not-for-profit basis in LMICs.

Ensuring distributive justice is not just about negotiation and contracting. Equal access to innovative medicine is a fundamental ethical demand that – at least regarding orphan genomic therapies – may require and justify a reorganisation of existing healthcare delivery systems.
